# An ecological study of temporal trends in ‘deaths of despair’ in England and Wales

**DOI:** 10.1007/s00127-022-02251-9

**Published:** 2022-03-05

**Authors:** Elizabeth Augarde, David Gunnell, Becky Mars, Matthew Hickman

**Affiliations:** 1 Population Health Sciences, University of Bristol, Bristol, UK; 2 Department of Health and Social Care, London, UK; 3 NIHR Biomedical Research Centre, University Hospitals Bristol and Weston NHS Foundation Trust, University of Bristol, Bristol, UK; 4 NIHR Health Protection Research Unit in Behavioural Science and Evaluation, University of Bristol, Bristol, UK; 5 NIHR School of Public Health Research, University of Bristol, Bristol, UK

**Keywords:** Epidemiology, Mortality, Suicide, Alcohol consumption, Drug use, Mental health

## Abstract

**Purpose:**

There is growing interest in the concept of ‘deaths of despair’ (DoD)—defined as deaths from three causes: suicide, drug poisoning, and alcohol-related conditions—as a more comprehensive indicator of the impact of psychological distress on mortality. The purpose of this study is to investigate the degree of commonality in trends and geographic patterning of deaths from these causes in England and Wales.

**Methods:**

WHO mortality data were used to calculate age-standardised, sex-specific temporal trends in DoD mortality and in mortality from suicide, drug poisonings, and alcohol-related conditions in England and Wales, 2001–2016. Three-year average crude rates were calculated for English local authorities for 2016–2018 and associations between rates were assessed using Spearman’s rank correlation.

**Results:**

Between 2001 and 2016, the DoD mortality rate increased by 21·6% (males) and 16·9% (females). The increase was largely due to a rise in drug poisoning deaths, with limited tracking between trends in mortality by each cause. DoD mortality risk was highest in middle-aged people; there were rises in all age groups except 15–24 year old males and 65 + females. There were strong positive correlations (*r* = 0.66(males) and 0.60(females)) between local authority-area drug poisoning and alcohol-specific mortality rates in 2016–2018. Correlations of these outcomes with suicide were weaker (*r* = 0.29–0.54).

**Conclusions:**

DoD mortality is increasing in England and Wales but there is limited evidence of commonality in the epidemiology of cause-specific mortality from the component causes of DoD (suicide, drug poisoning and alcohol-related conditions), indicating the need for tailored prevention for each outcome.

## Introduction

Economists Case and Deaton recently identified that a decades-long decline in mortality rate amongst middle-aged white non-Hispanics in the USA is reversing [1]. This phenomenon is largely attributable to increased mortality from suicide, drug and alcohol poisonings, and long-term alcohol-related conditions. They coined the term ‘deaths of despair’ (DoD) to describe this group of causes of death [2].

Suicide mortality is commonly used as an indicator of mental health, however, the concept of DoD centres on the notion that such intentional deaths underestimate the burden of despair on mortality. Trends in mortality by suicide may mask broader trends in deaths associated with despair and psychological distress, in part due to the inherent difficulty in determining intentionality in some deaths. Case and Deaton argue that drug and alcohol misuse are utilised as coping mechanisms for those experiencing distress brought on by socioeconomic or other circumstances, leading to deaths resulting from similar risk factors as suicides [2]. The aetiology of suicide, drug poisonings, and alcohol-related conditions are, therefore, similar because all are strongly associated with psychological distress [3–5].

There are concerning trends in each of these causes of mortality in the UK. Suicide mortality has been increasing since 2016 [6]. In contrast to many European countries, drug-related deaths in the UK continue to rise and constitute a public health crisis [7, 8]. Though some hypothesise that there are insufficient abstinence-based interventions [8], observational and modelling studies suggest that increasing coverage and retention into opioid agonist treatment is associated with reducing drug-related death rates [9, 10]. Liver disease mortality has been highlighted as a cause for concern in England, following a sustained increase in mortality since the 1990s, diverging from many European neighbours [11, 12]. Moreover, there is broad consensus that these outcomes are associated with deprivation [13–15] and contribute substantially to socioeconomic and geographical inequalities in mortality [3, 11, 13, 14, 16].

In the UK, recent attention has been drawn to rising mortality rates for some sub-populations and widening socioeconomic inequality in avoidable mortality [17], with brief reference made to the potential increasing role of DoD in middle-aged mortality [18]. Data from Scotland suggest there have been substantial increases in DoD in men aged 15–44, particularly since 2014 [19]. The present study builds on these findings by investigating DoD in both sexes and all ages in England and Wales. The primary aim of this study is to investigate sex-specific temporal trends in DoD mortality and in mortality from suicide, drug poisonings, and alcohol-related conditions in England and Wales. The secondary aim of this study is to investigate whether English local authorities experiencing high rates of one of the components of ‘deaths of despair’ (i.e. suicide, drug poisoning or alcohol-related mortality) also had higher levels of the other causes, as an indication of the consistency of these three markers of psychological distress. Evidence that mortality rates co-vary over time would support approaches to estimating the burden of mortality arising from mental and emotional distress and may support combined approaches to prevention.

## Methods

To achieve the primary aim, data for England and Wales were extracted from the World Health Organization (WHO) mortality database [20]. This database consists of deaths reported annually by WHO Member States since 1950. For England and Wales, data are supplied by the Office for National Statistics (ONS). Data were available for each International Classification of Diseases (Tenth Revision; ICD-10) [21] code by sex and 5-year age band for 2001–2016. Data were extracted for the ICD-10 codes used by Case and Deaton [1] to replicate their approach. We used the following ICD-10 codes for our analysis of mortality trends in England and Wales: Suicide: X60-X84, Y87.0; Drug poisoning: X40-X45, Y10-Y15, Y45, Y47, Y49; Alcohol: K70, K73, K74.

Annual age- and sex-specific mortality rates were calculated for suicide, drug poisoning, and alcohol-related mortality in 2001–2016 by combining the number of deaths for specific ICD-10 codes. The code groupings used replicate Case and Deaton’s analysis [1]. Age-standardised mortality rates (ASRs) were also calculated using ONS mid-year population estimates [22] and the 2013 European standard population. Combined annual DoD ASRs and ASRs for each individual ICD-10 code were also calculated. Rates and changes over time were tabulated and assessed graphically for all ages combined and 15–24, 25–44, 45–64 and  65+ year olds.

To address our second aim, we calculated average mortality rates for deaths from suicide, drug poisoning and alcohol in 2016–2018 for English local authorities. We used ONS mortality data obtained through the Public Health England Fingertips website [23] and ONS population estimates [22]. In this analysis, crude rates were calculated because age-specific data at this geographical scale was not available.

The following ICD-10 codes are used in ONS mortality definitions [24–26]: Suicide: X60-X84, Y10-Y34, Y87.0, Y87.2; Drug poisoning: F11-F19, X40-X44, X60-X64, Y10-Y14, X85; Alcohol: E24,4, F10,G31.2, G62.1, G72.1, I42.6, K29.2, K70, K85.2, K86.0, Q86.0, R78.0, X45, X65, Y15. These ICD-codes are different to those used in the England and Wales analysis of WHO data. There are also overlaps in the codes used in the ONS mortality datasets, meaning some deaths are counted in multiple datasets. Consequently, 19.7% of suicide deaths and 23.8% of drug poisoning deaths in England and Wales in 2018 are counted in both the suicide and drug poisoning death registrations data. 0.2% of suicide deaths and 0.1% of alcohol-specific deaths are counted in both the suicide and alcohol-specific death registrations data.

Local authorities were ranked according to 2016–2018 sex-specific mortality rate for each cause of death. Spearman’s rank correlation coefficients were calculated to assess the strength of the association between local authority ranks for each cause of mortality. Due to overlaps in the ICD-10 codes, findings from these analyses should be interpreted cautiously.

All analyses were completed in STATA version 15·0.

The data underlying this article were derived from sources in the public domain: WHO mortality database at https://www.who.int/data/data-collection-tools/who-mortality-database, ONS population estimates at https://www.ons.gov.uk/peoplepopulationandcommunity/populationandmigration/populationestimates, and Public Health England Fingertips website for local authority data at https://fingertips.phe.org.uk/

## Results

### Temporal trends in mortality from DoD, suicide, drug poisonings, and alcohol-related conditions in England and Wales (2001–2016)

**Fig. 1:**
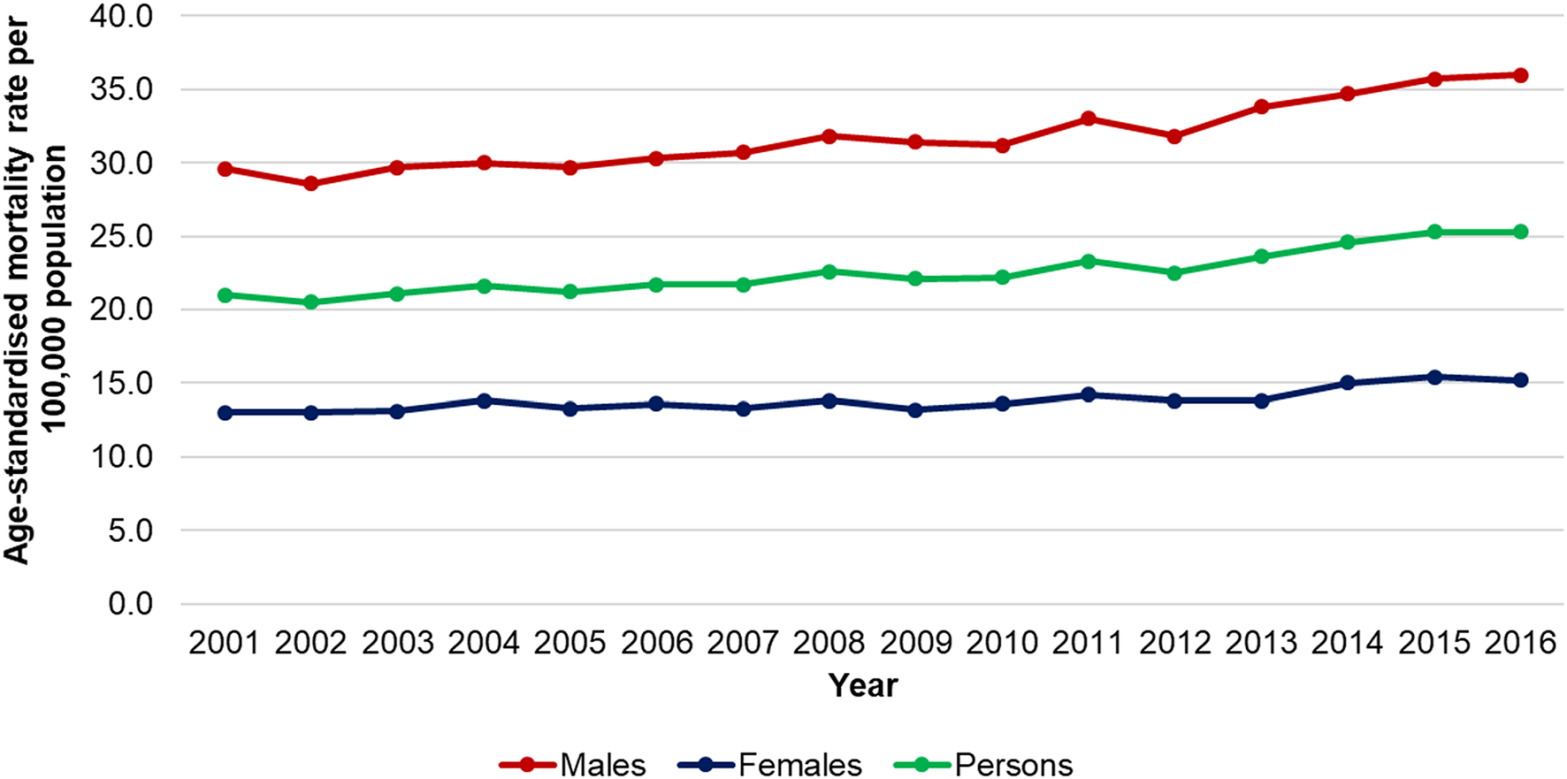
Age-standardised deaths of despair mortality rate, 2001–2016, England and Wales. Figure 1 shows the age-standardised deaths of despair mortality rate for males, females and persons on separate lines, for 2001–2016, for England and Wales

In 2016, 14,182 people died by either suicide, drug poisoning, or alcohol-related conditions in England and Wales. This equates to an ASR for DoD of 25·3 per 100,000 population (Fig. 1). DoD ASRs for both males and females increased between 2001 and 2016, by 21·6% for males from 29·6 to 36·0 per 100,000 population, and by 16·9% for females from 13·0 to 15·2 per 100,000 population.

**Fig. 2:**
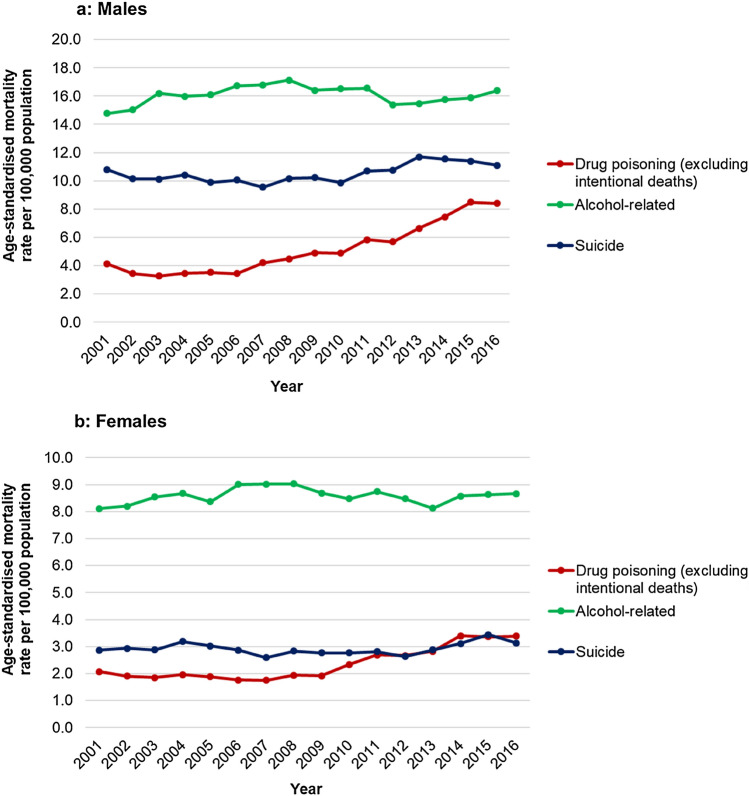
Age-standardised mortality rate by sex, 2001–2016 for component causes of death, England and Wales. Figure 2 is composed of two line graphs showing the age-standardised mortality rates for males (**a**) and females (**b**) for 2001–2016 in England and Wales. The graphs show the drug poisoning (excluding intentional deaths), alcohol-related and suicide mortality rates

The major contributor to DoD in both sexes was alcohol-related mortality, comprising 44.0% of male and 56.5% of female DoD in 2016. However, the increase in DoD over time was largely driven by a rise in deaths by drug poisoning (see Fig. 2a, b). Drug poisoning mortality increased by 104·9% for males from 4.1 to 8.4 per 100,000, and by 61.9% for females from 2.1 to 3.4 per 100,000. In contrast, between 2001 and 2016, mortality from suicide increased by 2·8% for males from 10.8 to 11.1 per 100,000, and by 6.9% for females from 2.9 to 3.1 per 100,000 (Fig. 2). Mortality from alcohol-related conditions increased by 10.8% for males from 14.8 to 16.4 per 100,000, and by 7.4% for females from 8.1 to 8.7 per 100,000. Trends in cause-specific mortality rates did not closely co-vary over time for either sex (Fig. 2).

The rise in alcohol deaths was largely due to an increase in liver disease (K70) mortality, which increased by 18.8% for males and 22.1% for females between 2001 and 2016. The increase in suicide deaths was not associated with a rise in any particular ICD-10 code. The increase in drug poisoning mortality was driven by a large increase in mortality rate for X42; deaths determined as being due to “accidental poisoning by and exposure to narcotics and psychodysleptics (hallucinogens), not elsewhere classified” [21]. This includes deaths caused by cannabis, cocaine, codeine, heroin, LSD, mescaline, methadone, morphine, and opium [21]. These accounted for 46.7% of male deaths and 13.2% of female deaths in 2001 but 57.8% of male and 34.7% of female deaths in 2016. These increases are substantially larger than for any other drug poisoning ICD-10 code. Notably, rates of the cause of death determination for drug-related suicide (X60-69) decreased by 24.6% for males and 38.9% for females over this time period.

The temporal pattern differed for different causes of death. Drug poisoning mortality rates show a sustained increase from around 2007 onwards, alcohol-related mortality rates changed relatively little over the study period (although there was a small increase overall), and suicide rates began to increase around 2010–12. These patterns were similar for both males and females.

### Age-specific temporal trends in mortality from DoD, suicide, drug poisonings, and alcohol-related conditions in England and Wales (2001–2016)

**Fig. 3:**
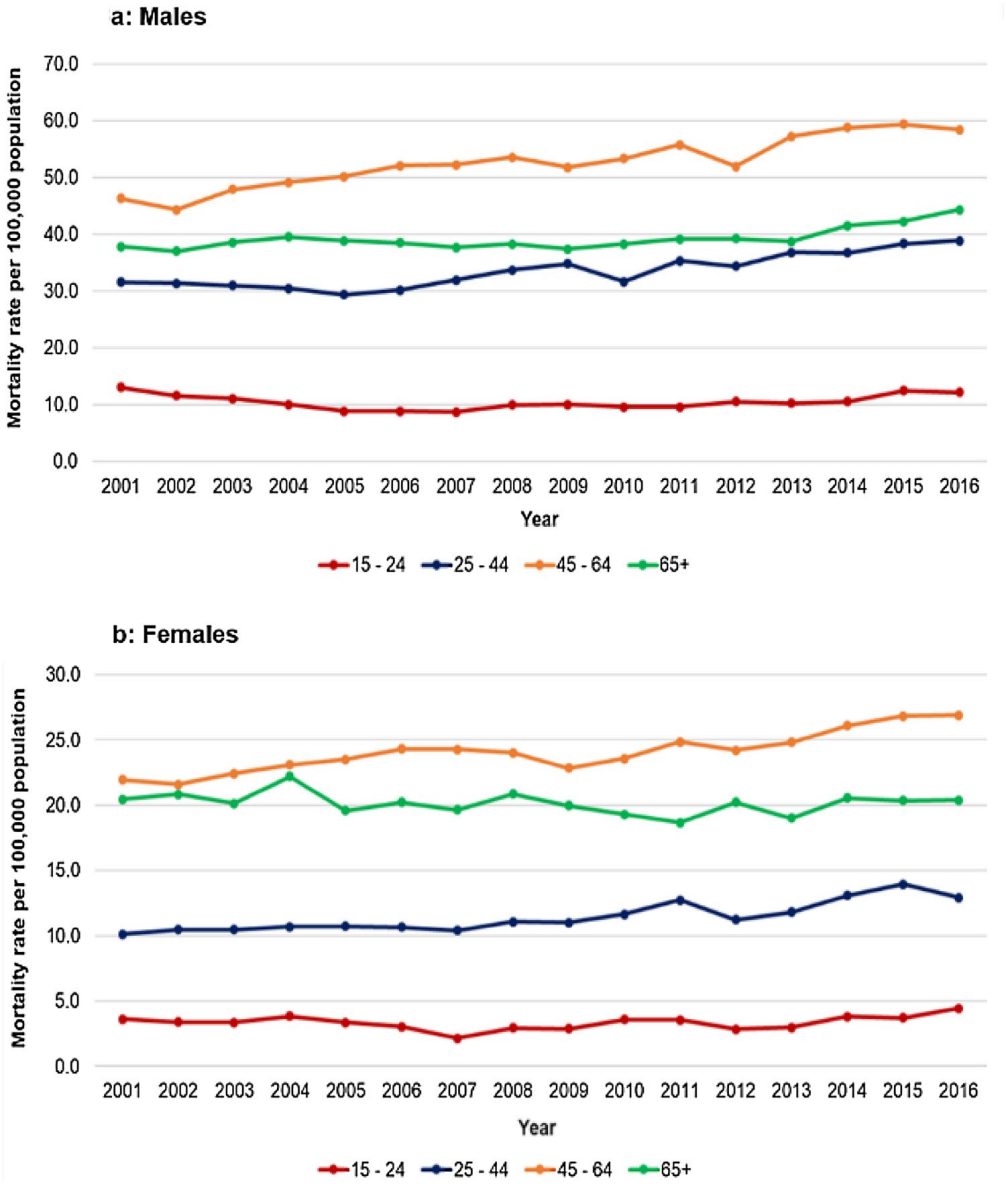
Age-specific trends in deaths of despair mortality rate, 2001–2016, England and Wales. Figure 3 is composed of two line graphs showing the age-specific deaths of despair mortality rates for males (**a**) and females (**b**) for 2001–2016 in England and Wales. The graphs show the mortality rates for ages 15–24, 25–44, 45–64 and 65 +

In both sexes, age-specific DoD mortality rate for those aged 45–64 is consistently higher than in other age groups, with the lowest rates experienced by those aged 15–24 (Fig. 3). DoD mortality increased over the study period in males of all ages except 15–24 year olds where there was a 6·9% decrease in rate from 2001 to 2016 (Fig. 3a). For females, DoD mortality rate for all age groups increased from 2001 to 2016 except those aged 65 + (Fig. 3b). The largest increase in male mortality rate was observed in those aged 45–64 (26·3%) whereas for females the largest increase was in those aged 25–44 (27·6%).

**Fig. 4:**
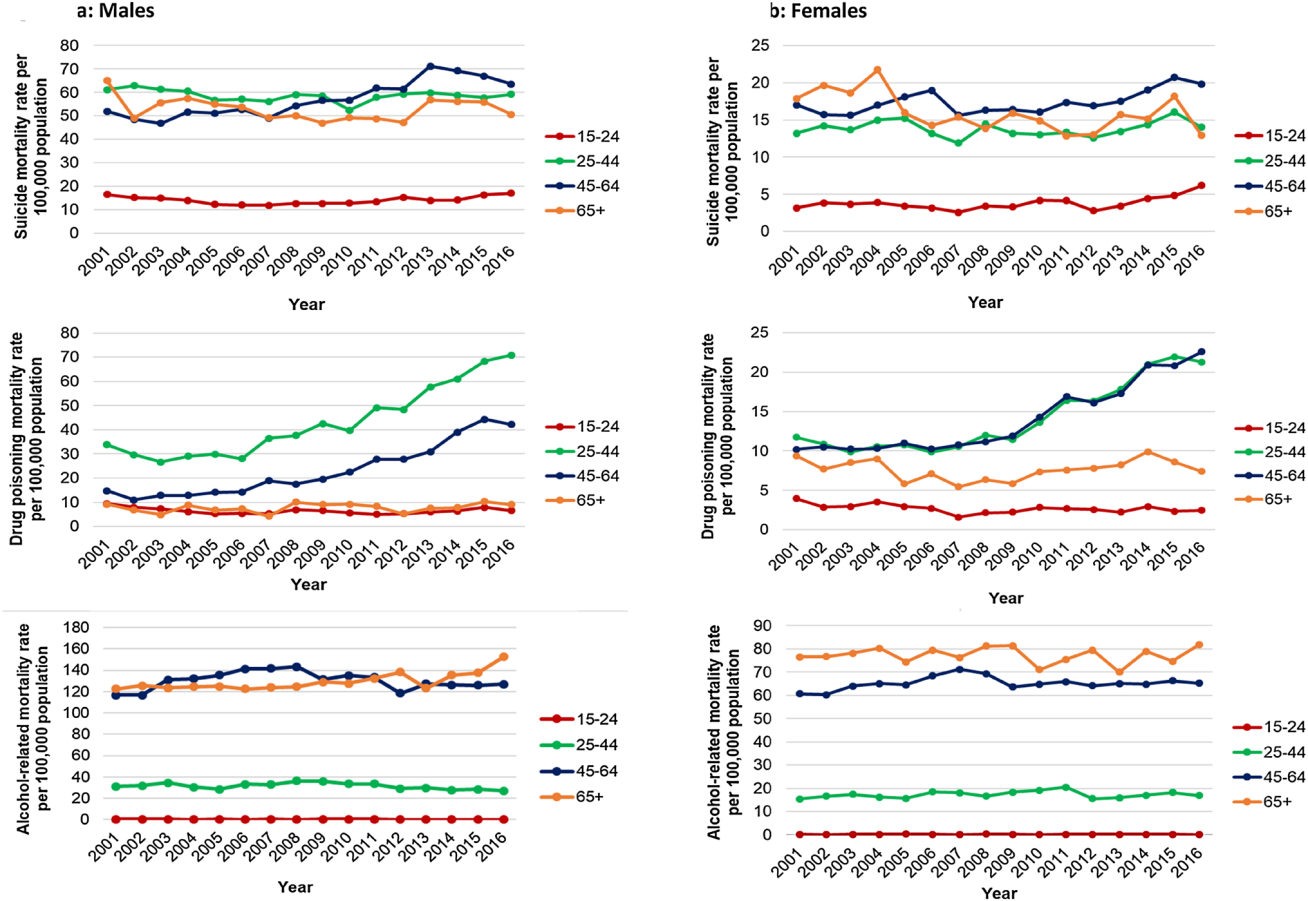
Age-specific mortality rates for suicide, drug poisoning and alcohol-related conditions, 2001–2016, England and Wales. Figure 4 is composed of six line graphs showing the age-specific mortality rates for males (**a**) and females (**b**) for 2001–2016 in England and Wales. For each sex there is a line graph for suicide, drug poisoning and alcohol-related mortality. The graphs show the mortality rates for ages 15–24, 25–44, 45–64 and 65 +

Male suicide rates increased slightly in those aged 15–24 and 25–44 from 2010, however, the largest change was observed in those aged 45–64, where rates increased substantially from 2001 to 2013, then declined (Fig. 4). Female suicide mortality rates for those aged 15–24 increased from 2012, with a similar pattern observed for ages 25–44 and 45–64. Female suicide rates in those aged 65 + fluctuated more since 2001, showing an overall decrease to 2016.

In both sexes, trends in drug poisoning mortality rates are also driven by large increases for those aged 25–44 and 45–64, with rates for those age 65 + and those aged 15–24 decreasing slightly over the same period (Fig. 4).

Male rates of alcohol-related mortality increased from 2013 amongst those aged 65 + and also increased slightly amongst people aged 45–64, whilst the younger age groups saw broadly stable rates over time (Fig. 4). The same patterns were observed in female rates of alcohol-related mortality, but the female mortality rate for those aged 65 + fluctuated more between 2001 and 2016.

### Variation in suicide, drug poisonings, and alcohol-specific mortality rates among English Local Authorities (2016–2018)

Considerable variation in all three outcomes is observed between local authorities in England. The interquartile range (IQR) of suicide mortality rates was 10.7 to 15.0 per 100,000 for males and 3.2 to 4.9 per 100,000 for females. The IQR of drug poisoning mortality rates was 6.3 to 11.8 per 100,000 for males and 2.8 to 5.5 per 100,000 for females. The IQR of alcohol-specific mortality rates was 10.4 to 18.8 per 100,000 for males and 4.7 to 9.3 per 100,000 for females.

For males, a moderate positive correlation was observed between the local authority ranks for suicide and drug poisoning mortality rates (*r* = 0·541, *p* < 0·01) and between ranks for suicide and alcohol-specific rates (*r* = 0·499, *p* < 0·01). A strong positive correlation is observed between alcohol-specific and drug poisoning mortality rates (*r* = 0·655, *p* < 0·01). The ICD-10 codes used in the alcohol-specific and drug poisoning datasets do not overlap.

Compared to males, in females there were weaker positive correlations between local authority ranks of suicide and drug poisoning mortality (*r* = 0.450; *p* < 0.01) and between suicide and alcohol-specific mortality(*r* = 0.287; *p* < 0.01); with the strongest correlation seen between alcohol-specific and drug poisoning mortality (*r* = 0.600; *p* < 0.01).

## Discussion

### Main findings

DoD mortality in England and Wales rose substantially between 2001 and 2016, with rates consistently higher for males than females. The rise is largely due to an increase in deaths from drug poisoning. Although, all causes of death (suicide, alcohol-related and drug poisoning) showed an increase over this time period, their trends did not closely co-vary over time at national level.

Alcohol-related mortality rates are highest throughout this time period, highlighting that alcohol misuse and related conditions are an important area for urgent intervention. Notably, alcohol use disorder is identified as a comorbidity in approximately 25% of suicide deaths [27]. Therefore, despite the fact this study finds minimal commonality between trends in suicide mortality and other outcomes, there may be important associations between conditions preceding each of these causes of death. It is also plausible that increases in suicide mortality rates may be followed after a time lag by increases in alcohol-related mortality, because alcohol-related conditions like liver failure develop gradually over time. In contrast alcohol intoxication may have an acute effect on mood and disinhibition arising from alcohol intoxication may increase the risk of a distressed individual acting on suicidal thoughts.

Case and Deaton’s research identified that increasing DoD mortality is a phenomenon primarily affecting middle-aged people, a finding reflected in this study and in research by Walsh and colleagues exploring DoD in birth cohorts in England [28]. Lived experiences in midlife appear to increase the risk of DoD, though it is unclear if this is a cohort effect. Low DoD mortality in those aged 15–24 may be because exposure to risk factors for emotional distress and DoD, such as unemployment, may not occur until later in life. Furthermore, many alcohol-related deaths may not occur until after many years of alcohol misuse.

We also investigated geographical variation in DoD. It is evident from the wide ranges in local authority mortality rates that there are substantial inequalities in suicide, drug poisoning and alcohol-related mortality associated with individual- or community-level risk factors for emotional distress. Intermediate factors influencing the likelihood of death, such as the availability of mental health and substance misuse services, may also be relevant. There was also evidence that local authorities with high levels of alcohol-specific mortality also had high rates death due to drug poisoning, but there were weaker associations (*r* = 0.29 to 0.54) between suicide mortality and these two outcomes. This indicates there may be some association at local level between drivers of alcohol-specific and drug poisoning mortality.

### What is already known

The findings of this study are consistent with recent work in Scotland, which reported an increase in DoD mortality rates, particularly since 2014, including very large increases in drug-related deaths [19]. Similarly, Walsh and colleagues identify similar age patterns in DoD mortality to those seen here [28]. Our findings also broadly reflect literature exploring trends over time in these causes of death separately [12, 13, 15, 29].

The trends observed here show some important differences to those identified by Case and Deaton among middle-aged white non-Hispanics in the USA [1]. In both countries, the rate of increase in drug poisonings was considerably greater than that observed for alcohol-related and suicide mortality [1]. However, Case and Deaton observed sustained increases each year from 1998, with minimal fluctuation year-on-year [1]. This may be the result of focusing on the middle-aged white non-Hispanic sub-population.

A concerning finding is the substantial increase in drug poisoning deaths in both sexes since around 2007. Case and Deaton hypothesise that increases in drug misuse mortality may in part be due to growing economic insecurity disproportionately affecting middle-aged whites who are driven to substance use as a coping mechanism [1, 2]. Our study provides some evidence that temporal changes in mortality may coincide with economic shocks, as mortality rates increased noticeably around the time of the 2008 financial crisis, particularly for drug poisoning and male suicides. US trends in DoD are thought to be linked to US drug policy as they coincide with the epidemic of opiate use, preceded by the US Food and Drug Administration’s 1998 approval of OxyContin as a prescription pain reliever and the marketing of OxyContin by large pharmaceutical companies [30–32]. England and Wales do not share the same policy history as the USA, though it has also experienced historic increases in opioid use in the population [33] with the increase in avoidable drug poisoning mortality raising a mixture of hypotheses around an ageing cohort and increased morbidity as well as failures in delivery of effective drug treatment [7, 9].

Furthermore, increased access to street fentanyl has been implicated in the increases in drug-related deaths in the USA [34, 35] whereas in the UK, fentanyl is still rarely implicated in UK drug deaths [36, 37] though other forms of poly-drug use with opioids have increased, such as street benzodiazepines and gabapentinoids [38, 39]. Future trends in drug poisoning mortality should be monitored closely for the possible impact of street fentanyl.

### What this study adds

To the best of our knowledge, this is the first epidemiological study of DoD in England and Wales. This study used publicly available data to generate important initial evidence for the value of exploring DoD and the commonalities between these causes of avoidable mortality in England and Wales, as a means of understanding the role of despair in premature mortality variation. Furthermore, our study adds further evidence for the high burden of alcohol-related mortality in England and Wales. Our findings also add to a growing body of evidence suggesting an urgent need for further research and intervention to understand and reverse the increasing trend in drug poisoning mortality.

### Limitations of this study

There are several limitations to our analysis. First, our findings cannot necessarily be generalised to other countries, as the policy contexts, economic circumstances and relative incidence of different component outcomes contributing to DoD may differ. Second, our assessment of trends over time was limited to a visual inspection of graphs; we did not undertake a more detailed time-series analysis to investigate commonality of trends and their associations with possible contributory factors such as changing economic indicators/periods of recession. The nature of the causes of death contributing to DoD may mean that trends of different indicators lag one another. For example, rises in alcohol misuse may not affect mortality for several years as conditions such as liver disease take several years to develop, whereas as acute distress, e.g. following financial difficulty and job loss, may immediately precede suicidal behaviour and death. Third, Case and Deaton’s analysis identified DoD as primarily a phenomenon affecting white non-Hispanics in the USA and highlights the inequality in DoD mortality between those with and without a college education [1, 2]. The influence of ethnicity and education status are not explored here but may be important in understanding differences in the findings of this study compared with Case and Deaton’s work—for example, in the USA, white non-Hispanics constitute only 60.1% of the total population [40], whereas in England and Wales they constitute 86.0% [41], and a larger proportion of the UK population have a degree compared to the USA [42, 43]. Fourthly, misclassification of deaths presents a possible issue for this study. Evidence shows that substantial misclassification of accidental overdose fatalities occurs in the USA [44] and in the UK [45]. Lastly, because of overlap in the ICD-10 codes used in our geographic analysis of cause-specific mortality, some of the correlations we observed are likely to be inflated, although of note, the strongest correlations were observed between alcohol and drug poisoning, where there is no overlap in codes.

A more fundamental limitation of our study relates to the conceptualisation of DoD which combines two causes of mortality stemming from acute incidents (i.e. suicide and poisoning) with deaths due to prolonged and accumulated exposure, such as alcohol-related liver disease. Though both the acute and chronic deaths relate to underlying chronic problems, such as addiction and chronic mental ill health, there will be differences in the lag between changes in the environment and the prevalence of risk behaviours, and time trends in DoD. These differences may be obscured through pooling. Nonetheless pooling also emphasises the importance of considering multiple related causes of death. It could also be argued that other causes of mortality associated with other adverse health behaviours, such smoking, should be considered in the definition of DoD.

### Research priorities

Future research could best utilise individual-level mortality data to investigate DoD according to Case and Deaton’s selection of ICD-10 codes at smaller geographical scales. This study suggests there may be substantial inequality in the burden of DoD. DoD may disproportionately affect smaller sub-populations, such as specific ethnicities, age- and socioeconomic groups [1], warranting further research into these inequalities and the potential for human-rights approaches to address the social determinants of psychological distress as a means of preventing DoD [46].

Furthermore, research utilising death certificate information would be required to determine which substances are behind increases in drug poisoning mortality. A Haddon’s matrix approach focusing on the structural and environmental risk has been highlighted as a possible approach to preventing harm from drug misuse and could usefully link such information with evidence from health and other sectors to characterise better opportunities for prevention [47–49]. ONS indicates that there has been a large increase in deaths involving opiates since 1993 [29]. In 2018, 51% of all drug poisoning deaths involved an opiate, most commonly heroin and morphine. Deaths involving cocaine have also increased, partly due to high purity levels [50]. However, the accuracy of these data is dependent on coroner-provided information, which is geographically and temporally variable [25, 45]. Indeed, 12% of drug poisoning deaths do not have a substance specified in the death record [25].

## Conclusion

DoD mortality is increasing in England and Wales with substantial inequalities in the risk of DoD mortality between age groups and localities. There is a clear need for research to better understand the epidemiology of DoD in the UK. Our findings provide further evidence of the continuing public health crisis in drug poisoning deaths in England and Wales and the need for more intensive and far-reaching interventions to reduce the number of drug poisoning and DoD deaths.
